# Giving advice to callers with mental illness: adaptation among telenurses at Swedish Healthcare Direct

**DOI:** 10.1080/17482631.2019.1633174

**Published:** 2019-06-27

**Authors:** Annica Björkman, Martin Salzmann-Erikson

**Affiliations:** a Faculty of Health and Occupational Studies, Department of Health and Caring Sciences, University of Gavle, Gavle, Sweden; b Department of Public Health and Caring Sciences, Uppsala University, Health Services Research, Uppsala, Sweden

**Keywords:** Complexity, nurses, organizations, qualitative, telemedicine

## Abstract

**Purpose**: Our aim was to describe Swedish Healthcare Direct (SHD) and its features as a complex system.

**Methods**: Qualitative interviews were conducted with 20 SHD telenurses, covering their experiences and skills when encountering and advising callers with mental illness. Complexity science was used as an *a priori* theoretical framework to enhance understanding of the complex nature of telenursing.

**Results**: SHD was described as a complex system as nurses were constantly interacting with other agents and agencies. During these interactions, dynamic processes were found between the agents in which the nurses adapted to every new situation. They were constantly aware of their impact on the care-seekers, and perceived their encounters with callers with psychiatric illness as “balancing on a thin line”. SHD was also described as both an authority and a dumping ground. The openness of the system did not give the nurses possibility to control the number of incoming calls and the callers’ intentions.

**Conclusions**: These new insights into SHD have important implications for organization developers and nursing management in terms of overcoming linear thinking.

## Introduction

Complexity science, otherwise known as complexity theory, is a well-established perspective within academic disciplines including economics, quantum physics, anthropology, cybernetics, sociology, psychology, meteorology, and nursing science. Stoehrel (2010) argued for its usability in studying dynamic systems that include behaviour of self-organization, such as human behaviour, cultures, and organizations. In accordance with other scholars (Coppa, 1993; Haigh, 2002; Mark, 1994; Ray, 1994; Walsh, 2000), we argue that it is appropriate to use complexity science in the field of nursing science and the study of healthcare organizations. Complex systems involve high levels of interaction, and are comprised of a large number of entities. Dugdale, Pavard, and Soubie (2000) explained that, in complex systems, it is very difficult to reduce the number of parameters or characterizing variables without losing its essential global functional properties. Conversely, using the term *a system* is somewhat misleading, as it appeals to an ontological idea of an autonomous entity. In order to deal with this paradox, Van Uden, Richardson, and Cilliers (2001) suggested the term “science of partial complex systems”.

**Table I.** Overview of the results.

Themes	Sub-themes
SHD as a complex system	Exposure of an open system to external processes
	Emergence of a pattern
Dynamic processes between the two agents	Nurses adapt to the situation
	Co-evolution
	Balancing on a thin line
	The flow of calls and interaction with other agents and systems
SHD as both an authority and a dumping ground	

Complex systems are interconnected and interdependent elements, but also contain feedback loops or processes that shape how change occurs within the system (Arnold & Wade, 2015; Gilli & Rossier, 1981). The features of complex systems also include emergence; that is, the way in which the behaviour of the system as a whole emerges from the interactions of different parts (Ramalingam, Jones, Reba, & Young, 2008). Complexity manifests itself through different phenomena, for example non-linear processes in which changes occur within the system. Changes are fundamental to complex systems, since small changes or differences in the initial conditions produce disproportionate changes in the outputs, a phenomenon known as *sensitivity to initial conditions* (Ramalingam et al., 2008). Due to the unpredictable conditions within a system, and given the condition that agents interact in a non-predetermined manner, complex systems are able to self-organize and spontaneously organize themselves without a controlling mechanism. Inhibition of such phenomena also exhibits an evolution over time. Thus, as adaptive agents interact, they co-evolve (Foster & Pyka, 2014; McDaniel & Driebe, 2001). In the present study, we ask how the telephone advice nursing service Swedish Healthcare Direct can be understood through the view of complexity science.

## Swedish healthcare direct

Telephone advice nursing (TAN) is a form of healthcare aimed at providing citizens with qualified nursing care and self-care advice, as well as steering patient flows and supporting care-seekers (Kaminsky, Rosenqvist, & Holmström, 2009, American Academy of Ambulatory Care Nursing, Greenberg, & Rutenberg, 2012). The TAN service in Sweden, Swedish Healthcare Direct (SHD), is an around-the-clock service offering advice and information 24 hours a day, 7 days a week, to the whole Swedish population of approximately ten million people. The approximately 1 300 nurses working within TAN (hereafter called “telenurses”) at SHD receive 4.5 million calls each year. The goal of SHD is to have short waiting times for care-seekers, and telenurses are expected to answer and document approximately 6–8 calls per hour. Previous research within the context of TAN has predominantly focused on telenurses’ and managers’ perceptions of TAN (Kaminsky, Carlsson, Holmström, Larsson, & Fredriksson, 2014; Purc-Stephenson & Thrasher, 2010), callers’ perceptions of the service (Williams, Warren, McKim, & Janzen, 2012), call characteristics, patient safety aspects (Ernesäter, Engström, Winblad, & Holmström, 2014; Leclerc et al., 2003), equity within TAN (Knowles, Munro, O’Cathain, & Nicholl, 2006), and communication between telenurses and callers (Ernesäter, Engström, Winblad, Rahmqvist, & Holmström, 2016). However, the everyday reality of nurses employed at SHD is a never-ending flow of incoming calls (Purc-Stephenson & Thrasher, 2010; Röing, Rosenqvist, & Holmström, 2013).

TAN is common in several Western countries such as the UK, the Netherlands, Australia, the US, and Canada. Nurses working within TAN are registered nurses working in call centres. Telenurses use an algorithm-based computerized decision support system (CDSS) to ensure quality of care (Huibers, Smits, Renaud, Giesen, & Wensing, 2011), provide uniform advice, and support them in their triage process (American Academy of Ambulatory Care Nursing et al., 2012). The CDSS mainly focuses on somatic symptoms of illness, and telenurses have previously described how they perceive the lack of information provided by the CDSS (Ernesäter, Holmström, & Engström, 2009). Telenurses handle queries and calls regarding all levels of symptoms from minor colds to chest pain. They never know what kind of call will be presented to them when they answer, and this forces them to always be prepared for the unexpected.

Reports show a negative trend of increasing psychiatric illness within the Swedish population, mainly among adolescents and older people (Bremberg, 2015). Psychiatric illness is an umbrella term which includes a wide range of conditions, from minor symptoms of anxiety to more complex mental illness such as schizophrenia and thoughts of suicide. This negative societal development demands preventive measures in order to avoid continuation of the trend, but stable resources within the healthcare system are also needed to assist, support, and counsel care-seekers with mental illness. Persons with mental illness are entitled to the same access to equal, knowledge-based, safe, and good-quality care as those with somatic illness (United Nations Human Rights Council, 2016). As well as being expected to answer many calls per hour, the performance of these telenurses is monitored and evaluated by the management. This call centre culture is problematic, since care-seekers with mental illness often require more time from the telenurses and need to be listened to (Bjorkman & Salzmann-Erikson, 2018). Telenurses also perceive the ethical dilemma of care-seekers potentially being lost in the healthcare system (Holmström & Dall’Alba, 2002; Holmström & Höglund, 2007). Within the UK, calls regarding mental disorder accounted for approximately 3% of total calls to NHS Direct; moreover, these calls were more complex and more time-consuming (mean time 23 minutes) than calls regarding somatic illnesses (Payne et al., 2003). Depressive problems and anxiety are the most common reasons for seeking help from telephone helplines (Payne et al., 2003; Sands, Elsom, Keppich‐Arnold, Henderson, & Thomas, 2016), and a majority of these calls are made by a relative (Payne et al., 2003).

## Rationale and aim

This study is part of a larger research project, “Telephone advice nurses’ complex work life in relation to callers with mental disorder”, conducted within the **Ch**aotic **a**nd Complex W**o**rk Life of Staff in the Healthcare **S**ystem (CHAOS) research group. Our studies so far have shown that telenurses experience problems when encountering care-seekers with mental illness problems, as they have nowhere to refer them (*cloaked citation during review process*). Telenursing is a complex form of nursing care (Snooks et al., 2008, Wahlberg & Bjorkman, 2018) which occurs within a controlled (Röing et al., 2013) and linear structure (Murdoch et al., 2015). The fact that the work procedure of TAN is guided by a CDSS (Ernesäter, Holmström, & Engström 2009) means that the nurses are obligated to reduce the caller’s symptoms and problems to something that can be documented within the structure of the CDSS (Murdoch et al., 2015). Telenurses have described how the CDSS might hinder and control their work (Ernesäter, Holmström, & Engström, 2009), and how safety threats related to the organisation contribute to decreased patient safety (Röing et al., 2013). The third most common cause for malpractice claims within TAN relates to organizational deficits (Ernesäter, Winblad, Engström, & Holmström, 2012). Moreover, we have found that the goals of the organization regarding expected work performance (expected number of calls per hour per nurse) collide with the expectations and needs of callers with mental disorders who desire the telenurses’ time and engagement. Although those studies provide insight into the daily clinical practice of telenurses, in this particular study our objective is to describe SHD and its features as a complex system.

## Methods

### Design

Due to the purpose of the study, a qualitative approach was adopted with a descriptive design. Part of our inspiration for conducting this study was a previous study in which Van Uden et al. (2001) addressed the suitability of using complexity science in the study of organizations.

### Settings and participants

We used a purposeful sample strategy (Polit & Beck, 2008) which allowed the participants to narrate their experiences of giving advice to callers with mental illness. At the time of the study there were 23 TAN services located all over Sweden. Based on differences between the TAN service centres e.g., size of centre (number of employees), geographic location (northern/southern country, large city/rural) and form of management (driven by county council/private management) five out of 23 centres were selected for the study. A research assistant with a masters’ degree in nursing science contacted the administrative managers at these five TAN services in Sweden, informed them about the research project, and asked for the work e-mail addresses of the nurses at that particular workplace. The research assistant sent an e-mail to all nurses employed at the respective TAN service centres (n = 5), one centre at the time, informing about the study. The nurses employed at the five TAN services received information about the study via e-mail, and the first nurses from respective TAN service (totally 20 nurses) who replied to the e-mail and met the inclusion criteria were included in the study. The inclusion criteria were 1) being a registered nurse and 2) having at least 12 months’ experience of working in TAN and 3) experience of encountering calls from care-seekers with mental illness during the last 12 month. Informed consent was given in writing prior to the interview.

### Data collection

In line with the qualitative approach, we used semi-structured interviews to construct the data material. Data were collected from May to October 2016 by a research assistant (also a registered nurse) with previous experience in qualitative interviewing. Participating telenurses were interviewed about their work life at SHD and their experiences and skills when encountering and giving advice to callers with mental illness. An interview guide was used, including questions such as “Please tell me about your experience of encountering and giving advice to callers with mental illness” and “How do you manage calls that include mental illness?” Follow-up questions were asked depending on the participant’s answer. Most interviews (19 of 20) were conducted via telephone due to the large geographical distances. The interviews were conducted during the participants’ regular work hours, and lasted a mean of 35 minutes. After transcription, the interviews comprised a total of 324 pages (Microsoft Word, Times New Roman, 12pt, 1.5 line spacing). The research assistant completed all interviews under the supervision of the researchers, which allowed us to become familiar with the data material.

### Data analysis

The steps in the descriptive analysis were guided by Sandelowski (2000). We engaged with the data material and read the interview scripts repeatedly. During the reading, words and sentences were extracted from the text and assigned codes which allowed us to find the “patterns or regularities in the data” (Sandelowski, 2000, p. 338). According to Sandelowski (1993), it is naïve to presume that any qualitative research can be approached *vis-à-vis*, and in the present study we committed to complexity science as an *a priori* theoretical framework to enhance understanding of the complex nature of telenursing (Sandelowski, 1993). As part of the coding process, all data analysis was conducted by both of the authors, who jointly discussed the content of the interviews in response to the codes and the pattern that emerged, and used these insights to write a tentative descriptive summary of the content. Recurrent discussions were carried out throughout the data analysis until consensus was achieved, and so all decisions regarding the analytical process were made jointly between the two authors. The flexible approach of qualitative description allowed us to revise the summary from our deepened discussion, thus moving from being immersed in the data to a more abstracted and interpretative understanding of the content. Both authors are registered nurses with previous work experience of mental health nursing (XX &YY, cloaked for review) and telephone advice nursing (XX). During the analysis, the research question guided us to ensure that the focus was maintained.

### Ethical considerations

The study was reviewed and approved by a Regional Ethical Board in Sweden (ref: xxxx/yyyy).

## Results

The results are reported in three themes and six sub-themes (see Table 1)

### SHD as a complex system

The first theme in the narratives described SHD as a complex system that involved interconnections and interdependencies with other systems, both on a larger and a smaller scale. On the smallest scale, the SHD system consisted of two interacting agents, the nurse and the caller, both of which were highly interconnected and entangled with other, larger systems. The nurse was highly interconnected with the SHD system as a whole; the workgroup of telenurses was a part of the workplace, which itself was a part of the national SHD organization, which in turn was a part of the Swedish healthcare system. Likewise, the caller was part of systems such as family and friends, work, and society. Thus, the caller’s narrative story was not grounded solely in the caller themselves, but also entangled in the caller’s surrounding systems, such as expectations from relatives and social stigma. This interrelationship between the small and the large was emphasized in the interviews, as the nurses referred to other healthcare providers (HCPs) to which they directed callers, for example emergency departments or primary care clinics. We interpret these descriptions as portraying TAN at SHD as a highly complex system. Variations within the descriptions are further described below in terms of the sub-themes.

#### Exposure of an open system to external input

SHD was seen as an open system due to its constant interactions with the surrounding systems. One example of external influences (input) mentioned by the participants was training provided by their employer, which was referred to in terms of a kick up the backside, a stimulus, or a renewal of knowledge (throughput). Some participants received further training on a regular basis, while others did not, but wished they did.

*Each month we have internal education. We make an education plan, and we’ve had psychiatric education. But, as you know, there are new staff and you lose it and … there’s a huge turnover of staff here*. (Interview 14)

External lecturers sometimes gave training at workplace meetings. The telenurses could then apply this external input in conversations with callers, and affect them in a health-promoting direction (output). This input-throughput-output is here interpreted as a reciprocal process between the internal and external environment of a system which ensures that the system survives and evolves.

#### Emergence of a pattern

The TAN service was an around-the-clock activity. The participants said that they continuously received calls from the public. However, in this ongoing flow of calls over which the nurses had no control (seeming chaos), it was possible to identify recurrences that formed a pattern, namely that calls addressing mental illness were predominantly received during the evening and night. Callers with psychiatric illness were perceived as fragile and more likely to misinterpret what was said. One participant said:

“[…] *we have some care-seekers who constantly call at night, and they get angry. You can’t do anything. So it’s really difficult.”* (Interview 10)

Another nurse, when asked about her experience of callers with mental illness, replied that

“It’s not that unusual, it isn’t. It isn’t so common during the daytime; they call at *night.”* (Interview 14)

Hence, the nurses saw callers with mental illness as shaping a pattern by contacting TAN outside of office hours.

### Dynamic processes between the two agents

The second theme was concerned with descriptions of the dynamic processes that took place between caller and nurse. The nurses needed to be sensitive and adapt to each situation, but in doing so they tried to steer the conversation in a certain direction by taking advantage of the inter-relational dynamics and trying to move forward. However, there was also uncertainty about the caller’s response, which could be seen as placing the dynamics on the edge of chaos.

#### Nurses adapt to the situation

The telenurses stressed the importance of being sensitive to the caller’s state of mind. Choosing the wrong word or making a “wrong” sound could damage the trust between caller and telenurse. The nurses paid extra attention to the caller’s reactions during the call, and were always prepared to adjust to the caller’s specific needs. Small “mistakes” could have a major impact on the relationship and on the outcome of the call (butterfly effect).

*When it comes to these people who are already fragile, and who are unwell, spiritually it’s like being more sensitive to the tone of voice and words, because a lot of the people we refer to psychiatric care call us back after a little while. And they say that it wasn’t very good, and they think they’ve been badly treated*. (Interview 13)

The participants did not usually consider calls regarding psychiatric illness to require immediate care. They said they took a more administrative role towards these callers, rather than a caring approach, and focused on providing them with accurate information such as phone numbers and contact information. They perceived callers as autonomous care-seekers capable of handling their own situation.

*But it’s rarely an emergency … But otherwise it feels like, and it’s true, too, I can’t, like, take over, these are fully, really, fully functioning individuals who can handle this, really*. (Interview 3)

Other aspects of adaptation were found in the participants’ descriptions of how they had to be constantly ready to adapt to the situation presented by the care-seeker. They had to switch from handling simple calls about minor problems, such as colds and flu, to dealing with life-threatening conditions. Moreover, care-seekers whose calls were initially about minor problems could suddenly change the subject to thoughts of suicide. The nurses had to adapt to this process and always be prepared to encounter calls from care-seekers who wanted to end their life or from relatives who had found a close family member taking an overdose.

*Because it’s all so complex, like I’m telling you, it might go from being a common ailment to someone who’s considered suicide by the end of the conversation, like it can just eventually pop out of them*. (Interview 20)

Calls were described as changing during the course of the conversation; for example, an aggressive caller might become calm and express a death wish during the call, as the telenurse let them express their feelings and listened to their despair.

#### Co-evolution

The participants described how they tried to incite a change in the care-seeker’s way of viewing their situation by supporting them, by providing them with the right amount of correct information, and by not being exaggeratedly positive in order to help the care-seeker see their situation from a different perspective. This constituted a kind of co-evolution. They felt that the anonymity of the telephone enabled them to hear life stories that would not be facilitated by a physical face-to-face encounter. The distance created by the telephone was thus used to enable a close and honest care situation.

#### Balancing on a thin line

The participants said they felt as if they were balancing on a thin line between helping care-seekers versus taking over their autonomy, while at the same time adjusting to the individual’s needs. This was described as contributing to the complexity of TAN. This complexity had increased over the years, and today’s care-seeker was perceived to have more complex psychiatric illness with a combination of medical diagnosis, drug abuse, social problems/dysfunction, and language problems. The telenurses described how they felt unable to add anything of value to the situation of these care-seekers.

“*But I mean back then it wasn’t so complex. I mean, nowadays these people with mental problems often have mixed substance abuse to start with, and then they haven’t received their diagnosis, I mean there’s so much of that now that’s new, so. So a lot has happened.”* (Interview 4)

#### The flow of calls and interaction with other agents and systems

The nurses often talked about being interconnected and interdependent with other systems in their work. For example, in case of emergencies and other severe situations they referred calls to SOS Alarm 112 (an emergency telephone line equivalent to 999 in the UK or 911 in the US), and in less acute situations they directed and guided callers to specific clinics.

*My job is to identify what the main symptoms are, really, and how I can best help them, and refer them on, and so on*. (Interview 2)

Although the participants acknowledged that referring the caller to another part of the healthcare system was a major task, they also noted that they did not refer all calls associated with mental illness to the psychiatric emergency unit.

### SHD as both an authority and a dumping ground

The participants described a change in the perception of SHD among the surrounding healthcare systems. One said that many other healthcare services increasingly referred to SHD as being a kind of oracle which was able to answer all kinds of questions, no matter the context or the kind of expertise required. The result of many agents indirectly interacting with each other via patients was that the system evolved into new formations. This evolution was not an entirely advantageous one, since it had ignited the process of degrading SHD into what was expressed as a dumping ground.

*Something I also think is a pity, it’s that so many of them have started referring people to SHD. It’s become a bit of a catch-all, if I may say so. Because there’s always someone here to answer the phone, there’s always someone here (ironic tone), and we usually manage to solve everything*. (Interview 9)

The participating telenurses believed that changes among other HCPs, such as decreased resources within psychiatric care together with an increase in the prevalence of psychiatric illness within the community, led to an increase of SHD callers with psychiatric disorders. However, due to limited resources of time, the complex nature of these calls did not fit with the desires of the nurses’ managers. The managers wanted the nurses to conduct short and effective calls, while the callers wanted the nurses to take the time to listen and to help them. The nurses perceived that many callers regarded SHD as their last chance for help, and held high expectations of the telenurses’ ability to help them which the telenurses were unable to fulfil due to organizational goals. We interpret this as a lack of space for the caller’s narrative, which created a disharmony between the caller’s anticipation and needs and the telenurse’s preconditions. Based on the narratives, our interpretation is that this disharmony may lead to feelings of guilt and moral distress among the telenurses.

## Discussion

The aim of this study was to describe SHD as a complex system and its features in relation to callers with mental health issues. The results indicate that SHD is constantly interconnected and interdependent with the surrounding systems, and does not exist in a vacuum. As previous scholars have noted, studying healthcare organizations from a complexity perspective is highly relevant (Coppa, 1993; Haigh, 2002; Mark, 1994; Walsh, 2000). However, no previous study has specifically analysed SHD from a complexity perspective. Our results demonstrate SHD to be a dynamic and self-organizing organization based on the constant involvement of human interactions. Nevertheless, our study also shows that these telenurses used different measures to try to create order in the chaotic worlds of their callers, which in turn affected SHD as a system. Smaller changes among other HCPs spread rapidly to SHD via a butterfly effect, and the telenurses had no option but to adjust and adapt, with no possibility to affect the situation. The nurses were constantly aware of the impact they had on the care-seekers, and encountering callers with mental illness. We interpret this as their “balancing on a thin line”, which is a contextualization of the “edge of chaos concept frequently emphasized in complexity science (Carroll & Burton, 2000, Ramalingam et al., 2008). The nurses’ verbal and nonverbal responses had a tremendous impact on the rest of the call, as a misplaced word or sound could have a butterfly effect on the future interaction between the parties. Our findings make it possible to acknowledge that nurses at SHD would be able to improve the quality of care via education, guidelines, and a more detailed decision-based system. As Stoehrel (2010) explains, the goal of system theory is to seek equilibrium within the system. In contrast, complexity science acknowledges the positive feedback loops which can place a system into a non-equilibrium state and push it to evolve. Hence, we argue that the complexity perspective provides SHD with greater opportunities to improve the quality of care for both agents; the callers and the nurses. Controlled inputs that seek evolution are generally accepted and understood, but inputs may also be uncontrolled in terms of the unpredictable, which might be more difficult to cope with. Concerning complexity thinking, change is a process in which the process itself is affected by unknown and unpredictable events. Learning is a constant factor throughout this, and corresponds to what is termed co-evolution in complexity science. Our results show that the callers were affected by the nurses’ input and that changes occurred during the course of the conversation, and so the two systems of the nurses and callers had coevolved. The telenurses described how the care-seekers with mental illness issues did not fit into the managerial expectations of the organization, which produced a collision between needs and possibilities. Deady and McCarthy (2010) argue that if caregivers are not given the opportunity to adequately address care-seekers’ concerns, the quality of decision-making might be impaired.

Managers are aware of the complexity of TAN, and describe multitasking as one of its more specific challenges (Kaminsky et al., 2014). However, the results of the present study imply that linear thinking permeates the view of TAN by system developers (of the CDSS) and managers. This linear thinking engenders interference with the complex reality of the nurses’ work situation, for example being controlled, monitored, and assumed to answer a certain number of calls (Röing et al., 2013). Thus, we argue that this study clearly demonstrates that TAN is a complex system which requires a cohesive view from developers to end users. Another example of the linear thinking within SHD is the organization’s response to malpractice claims; measures taken by SHD most commonly involved discussion in a work group and education of staff. Despite the severity of the cases, the measures taken mainly addressed active failure (Reason, 2004) rather than the latent conditions contributing to the error (Reason, 2000). Telenurses who had been involved in serious malpractice claims (Röing & Holmström, 2015) described how situations over which they had no control made them stressed and contributed to the malpractice claims. Stressful work, irregular work hours, cognitive fatigue, problems with understaffing, and system factors have also been shown to affect patient safety within TAN (Kaminsky, Röing, Björkman, & Holmström, 2017).

Telenurses at SHD are dependent on other HCPs to perform their work. The problem of limited accessibility among other HCPs has been previously described (Ernesäter, Engström, Holmström, & Winblad, 2010); this is not a problem local to Sweden, but is also a growing problem internationally. If HCPs attempt to resolve availability problems by referring patients to SHD, the problem is only moved, and not solved. Telenurses have previously described how they often do not know where to refer a care-seeker they have encountered (Holmström & Dall’Alba, 2002). In the UK, vulnerable people have been shown to be less likely to contact TAN services (Knowles et al., 2006). To our knowledge, there are no previous studies examining TAN from the standpoint of complexity science, but other studies regarding equity within TAN have shown that vulnerable patient groups are at risk of not receiving appropriate care (Holmström & Höglund, 2007; Knowles et al., 2006). Mental illness is increasing in today’s society, especially within the younger population, which implies that the perceived problem of decreased resources is a potentially large problem that needs to be solved. Care-seekers with mental illness constitute a vulnerable group, and so this problem should be addressed and measures taken to ensure this population of patients is given the appropriate care to which they are entitled.

On the macro level of interaction between different professionals such as other HCPs and other telenurses, studies have shown that telenurses feel questioned and belittled by other HCPs but find support from fellow telenurses (Ernesäter et al., 2010). On the micro level of interaction between the two agents, studies have shown how the interaction between telenurse and caller might be disrupted by external factors such as the CDSS (Ernesäter et al., 2009), mutual perceptions of the other actor’s credibility (Höglund & Holmström, 2008; Hansen & Hunskaar, 2011; Kaminsky et al., 2014;), and the fact that telenurses are exposed to specific organizational goals such as preferred call times (Röing & Holmström, 2015). The care-seeker’s gender has also been shown to be of importance, as telenurses have reported that they did not trust the competence of fathers who called, having greater trust in the mothers (Höglund & Holmström, 2008).

### Methodological considerations

Our interviews were focused on descriptions of encountering callers with mental health issues, and so one possible limitation is that the system of SHD was not fully represented, since far from all calls involve mental illness. However, we argue that acquiring data from nurses’ descriptions of their work in general would not have provided insight into how the system reacts when exposed to the unexpected. Several measures were taken to enhance trustworthiness of the findings (Graneheim & Lundman, 2004). To strengthen credibility, the analysis was performed by both authors (both RNs), and recurrent discussions regarding the meaning units and their subsequent categorizations were carried out throughout the analysis until consensus was achieved. To further strengthen credibility, quotations have been presented in the results section. To strengthen dependability, all interviews were performed by the same person using a semi-structured interview guide which was tested in a pilot interview. To further strengthen dependability, we chose participants who varied in aspects such as size of health centre, geographic location, and form of management.

### Conclusion

Our findings provide insight into SHD as a complex system based on its features, its ability to adapt and change, and its agencies. This study raises important questions about the nature of SHD in terms of being an open system/organization that is interconnected and interdependent with surrounding systems. These findings enhance our understanding of SHD and its fragility; even a small change can affect the system as a whole, in a way similar to the butterfly effect. In this sense, we conclude that telenurses have no option but to adjust and adapt, with no possibility to fully control the situation. However, telenurses are constantly aware of their impact on care-seekers, and perceive encounters with callers with mental illness as “balancing on a thin line”.

## Abbrivations

CDSS: Computerized decision support system
CHAOS: The Chaotic And Complex Work Life of Staff in the Healthcare System
HCP: Healthcare provider
SHD: Swedish Healthcare Direct
TAN: Telephone advice nursing

## Availability of data and supporting materials

Please contact the corresponding author for data requests.
